# Continuous or extended vs intermittent infusions of beta-lactam antibiotics in ICU patients with pneumonia: a systematic review and meta-analysis of randomized controlled trials

**DOI:** 10.1128/aac.00732-25

**Published:** 2025-08-27

**Authors:** Yixuan Li, Jason A. Roberts, Mohd H. Abdul-Aziz, Fekade B. Sime

**Affiliations:** 1 University of Queensland Centre for Clinical Research, Faculty of Medicine, The University of Queensland 420004, Brisbane, Australia; 2 Herston Infectious Diseases Institute (HIDI), Metro North Health, Brisbane, Australia; 3 Department of Pharmacy, Royal Brisbane and Women's Hospital 3883, Brisbane, Australia; 4 Department of Intensive Care Medicine, Royal Brisbane and Women's Hospital, Brisbane, Australia; 5 UR UM 103, Division of Anesthesia Critical Care and Emergency and Pain Medicine, Nimes University Hospital, University of Montpellier, Nimes, France; Providence Portland Medical Center, Portland, Oregon, USA

**Keywords:** beta-lactams, continuous infusion, extended infusion, prolonged infusion, ICU patients, pneumonia

## Abstract

This systematic review and meta-analysis of randomized controlled trials (RCTs) of intensive care unit (ICU) patients with pneumonia was conducted to compare the clinical outcomes of beta-lactam antibiotics when administered by prolonged infusion vs intermittent infusion. The systematic search was conducted in Medline (via PubMed), CINAHL, EMBASE, Cochrane Central Register of Controlled Trials (CENTRAL), and ClinicalTrials.gov. The outcomes were mortality, clinical cure rate, microbiological cure rate, adverse events, and ICU length of stay. The pooled risk ratios or mean differences were estimated by the fixed or random effect methods according to heterogeneity statistics. Twelve eligible RCTs were included in the meta-analysis. ICU length of stay was lower in the prolonged infusion patients compared with intermittent infusion. However, compared to intermittent infusion, the prolonged infusion, both continuous and extended infusion, of beta-lactam antibiotics was not associated with statistically significant improvements in mortality, clinical cure rate, microbiological cure rate, and rate of adverse events. For the treatment of ICU patients with pneumonia, prolonged infusion was associated with a shorter ICU length of stay. Multicenter RCTs with large sample sizes of ICU patients with pneumonia are needed to better assess other important endpoints.

## INTRODUCTION

Pneumonia is one of the most common nosocomial infections in the intensive care unit (ICU), with reported morbidity ranging from 6.8% to 68% (1, 2) and high mortality rates between 20.9% and 65% (2–4). In addition, ICU patients with nosocomial pneumonia usually have a longer duration of hospitalization and ICU length of stay, causing a significant economic burden to both patients and the healthcare systems (5–7). If a patient develops nosocomial pneumonia, appropriate empirical antibiotic therapy should be initiated early to maximize clinical outcomes (8, 9).

Beta-lactam antibiotics are widely used for initial empiric antibiotic therapy for pneumonia owing to their broad spectrum of activity (10). These antibiotics exhibit time-dependent bactericidal activity such that the duration for which the target exposure (concentration at the site of infection) exceeds the MIC of the causative pathogen is the critical determinant of efficacy. This is usually measured as the percentage of the dosing interval that the concentration of free drug remains above the MIC (%*f*T_>MIC_) (11). An exposure target of 40%–70% *f*T_>MIC_ is generally considered sufficient for beta-lactam antibiotics. For critically ill patients, the recommended pharmacokinetic/pharmacodynamic (PK/PD) target was %*f*T_>4MIC_ = 100% (12). However, due to epithelial lining fluid penetration that is frequently incomplete, as well as disease-induced pharmacokinetic alterations of beta-lactam antibiotics in critically ill patients, standard intermittent infusion regimens may fail to achieve these exposure targets in ICU patients with pneumonia (13, 14). Several studies have demonstrated that prolonged infusion of beta-lactam antibiotics is an effective strategy to increase %*f*T_>MIC_ exposure (15–18).

Several clinical studies have independently reported the benefits of prolonged infusion compared to intermittent infusion of beta-lactam antibiotics in pneumonia patients (15, 19–24). However, data specifically focused on pneumonia patients remain limited. In 2016, Lal et al. reported that there was no significant difference in mortality (odds ratio [OR] 0.85, 95% confidence interval [CI] 0.63–1.15) between prolonged vs intermittent infusion groups based on a meta-analysis of 10 studies (5 randomized controlled trials [RCTs] and five retrospective studies) (25). However, some studies published in non-English languages were not considered, and some additional large RCTs have been conducted since then.

The aim of this systematic review and meta-analysis was to compare the clinical outcomes of prolonged vs intermittent infusions of beta-lactam antibiotics in ICU patients with pneumonia.

## RESULTS

### Summary of included studies

**Fig 1:**
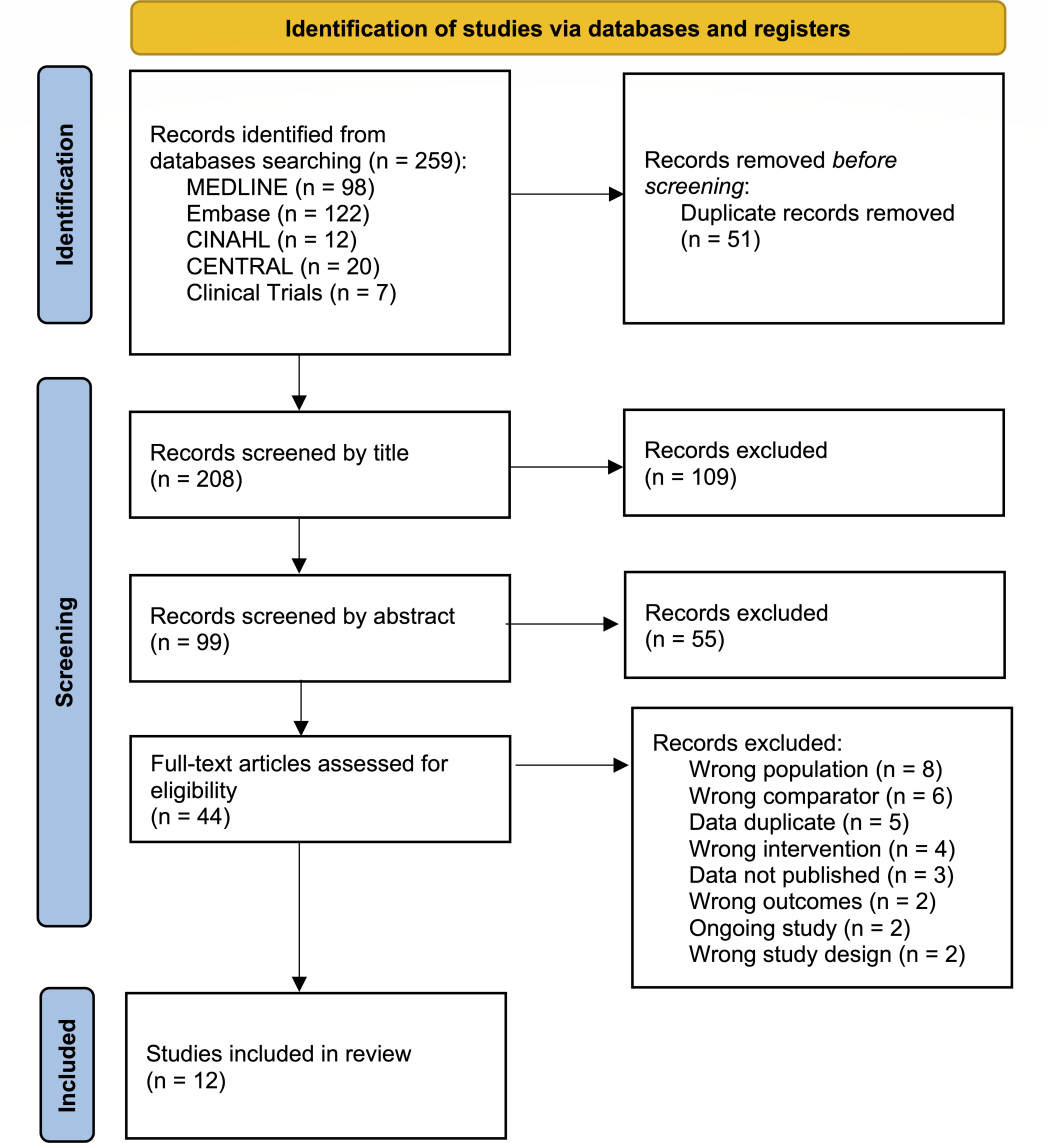
Preferred Reporting Items for Systematic Reviews and Meta-Analyses (PRISMA) flow diagram of study selection.

Twelve studies with 1,049 participants were identified for inclusion in our analysis. Figure 1 shows the study selection process.

A description of the included study characteristics is summarized in Table 1. Of the 12 RCTs, 5 compared continuous vs intermittent infusion, and 7 compared extended vs intermittent infusion of beta-lactams. The included studies were conducted in various countries, including China (*n* = 4), the USA (*n* = 2), Belgium (*n* = 1), Czech (*n* = 1), Egypt (*n* = 1), France (*n* = 1), Germany (*n* = 1), and multi-country (*n* = 1), with the year of publication ranging from 2000 to 2018.

**TABLE 1:** Characteristics of included studies^*a*^

Study	Duration and region	Sample size	Gender (M/F)	Age (mean)	Type of pneumonia	APACHE II (mean)	Antibiotics	PK PD target attainment (*f*T_>MIC_, C_min>4×MIC_, or T_>MIC_)	
								PI	II
Cousson (20,20)	NI, France	CI: 17 II: 17	CI: 13/4 II: 14/3	CI: 70 II: 61	VAP	NI	CAZ	NI	NI
Hanes (26)	NI, USA	CI: 16 II: 14	CI: 14/4 II: 11/3	CI: 33.5 II: 36.1	NP	CI: 12.8 II: 10.9	CAZ	100	>92
Li (27)	Jan/2006–Dec/2008, China	CI: 32 II: 34	CI: 18/14 II: 21/13	CI: 51.4 II: 50	CAP	CI: 16.8 II: 17.33	TZP	NI	NI
Nicolau (28)	NI, USA	CI: 19 II: 18	CI: 10/7 II: 13/5	CI: 46 II: 56	NP	CI: 13.9 II: 15.5	CAZ	NI	NI
Sakka (29)	NI, Germany	CI: 10 II: 10	CI: 6/4 II: 5/5	CI: 62 II: 59	VAP	CI: 26 II: 28	IMI	NI	NI
Ammar (30)	Oct/2015–Dec/2016, Egypt	EI: 30 II: 30	EI: 24/6 II: 23/7	EI: 55.8 II: 55.5	VAP	EI: 19 II: 20	MEM	NI	NI
Frippiat (31)	Jan/2012–Sep/2012, Belgium	EI: 25 II: 30	EI: 18/7 II: 17/13	EI: 65.7 II: 61.5	NP	NI	MEM	100%	100%
Lipš (32)	NI, Czechia	EI: 10 II: 9	EI: 9/1 II: 5.4	EI: 63 II: 57	NP	EI: 26 II: 29	IMI	85.15%	87.4%
Lü (33)	Mar/2012–Oct/2012, China	EI: 25 II: 25	EI: 14/11 II: 15/10	EI: 69.75 II: 67.04	NP	EI: 23.17 II: 23.73	TZP	86.82%	42.84%
Merchant (34)	Jun/2004–Oct/2006, Multi-country	EI: 249 II: 251	EI: 195/54 II: 192/60	EI: 51.4 II: 51.7	VAP	NI	DPN/IMI	NI	NI
Wang (35)	Mar/2006–Jul/2006, China	EI: 15 II: 15	EI: 10/5 II: 9/6	EI: 44.33 II: 39.67	NP	EI: 20.33 II: 17.33	MEM	75.3%	54%
Wang (36)	Sep/2012–Sep/2013, China	EI: 38 II: 40	EI: 25/13 II: 34/16	EI: 63.5 II: 57.3	NP	EI: 20.7 II: 19.2	MEM	NI	NI

^*a*^ PI, prolonged infusion; II, intermittent infusion; NI, no information; CI, continuous infusion; EI, extended infusion; NP, nosocomial pneumonia; VAP, ventilator-associated pneumonia; CAP, community-acquired pneumonia; MEM, meropenem; CAZ, ceftazidime; TZP, piperacillin/tazobactam; IMI: imipenem/cilastatin; DPN: doripenem.

### Risk of bias of included studies

The details of the risk of bias assessment by the RoB 2 tool of included RCTs are presented in supplementary information S5 at GitHub. A comprehensive assessment was conducted in five domains (randomization process, deviation from the intended interventions, missing outcome data, measurement of the outcome, and selection of the reported result). The overall risk of bias analysis was not highly biased, but certain aspects raised some concerns. No significant publication bias was observed in both funnel plots and Egger’s test results (supplementary information S6 at GitHub).

### Mortality

**Fig 2:**
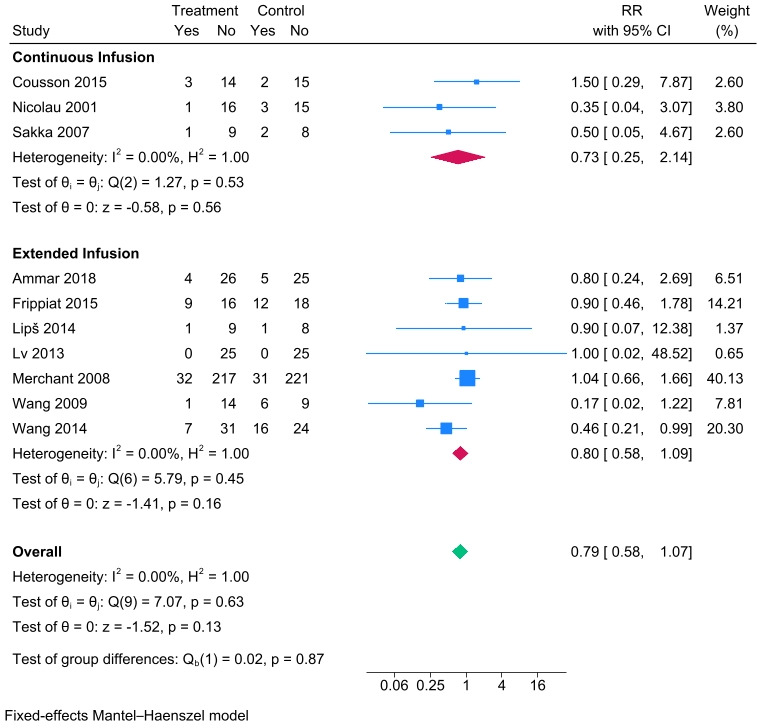
A forest plot representation of the comparison of the mortality rate between prolonged infusion vs intermittent infusion of beta-lactam antibiotics.

For mortality, 10 prolonged vs intermittent infusion RCTs (20, 28–36) were included. Compared to the intermittent infusion group, the mortality of the prolonged infusion group was lower but not significantly (Fig. 2, risk ratios [RR] 0.79, 95% CI 0.59–1.07). The same trends were observed in both subgroups of continuous infusion (*N*_CI_ = 44, *N*_II_ = 45, Fig. 2, RR 0.73, 95% CI 0.25–2.14) and extended infusion (*N*_EI_ = 392, *N*_II_ = 401, Fig. 2, RR 0.8, 95% CI 0.58–1.09).

### Clinical cure rate

**Fig 3:**
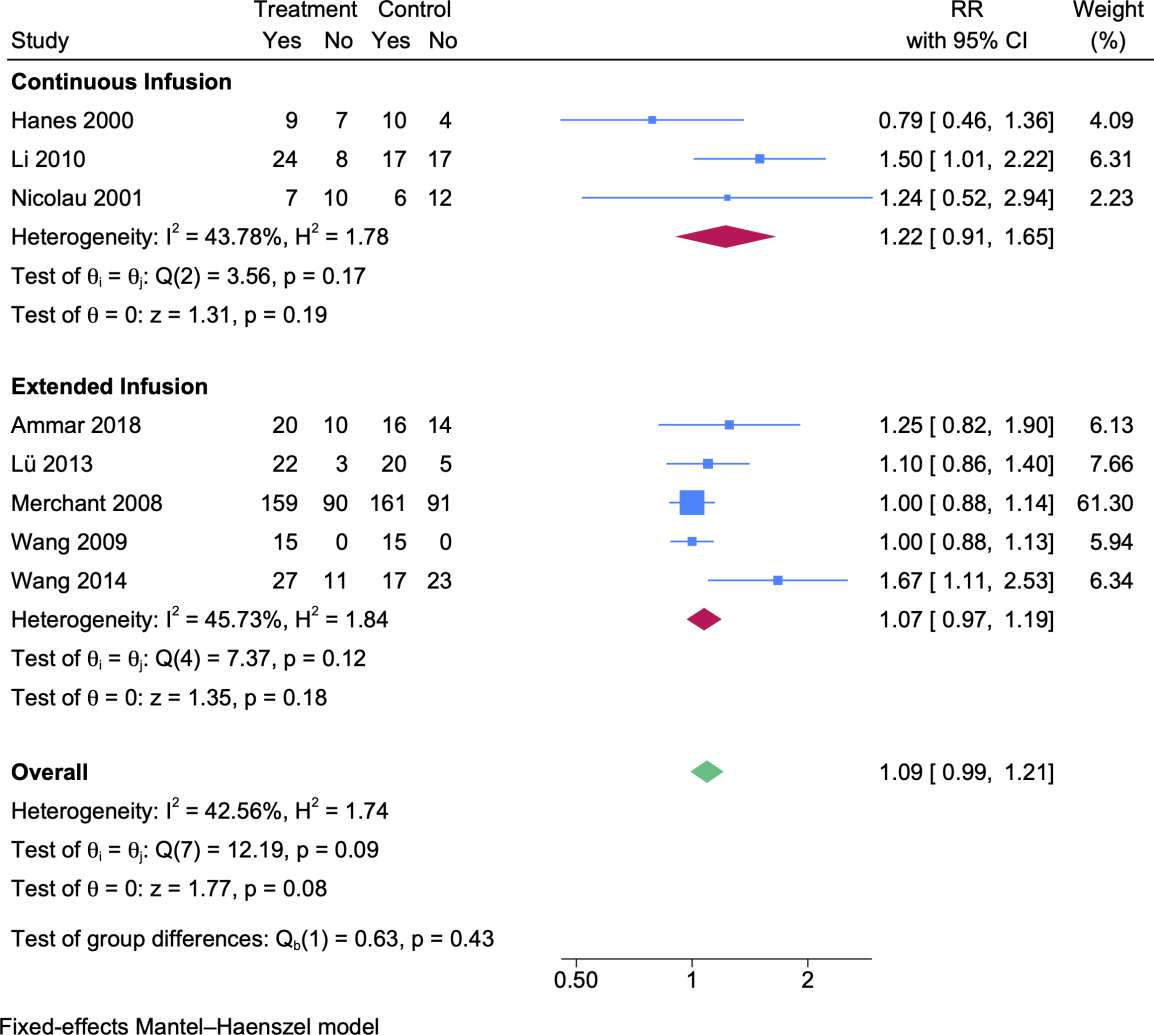
A forest plot representation of the comparison of the clinical cure rates between prolonged infusion vs intermittent infusion of beta-lactam antibiotics.

For clinical cure, eight prolonged vs intermittent infusion RCTs (26–28, 30, 33–36) were included. Compared to the intermittent group, the clinical cure rate of the prolonged infusion group was higher but not significantly (Fig. 3, RR 1.09, 95% CI 0.99–1.21). The same trends were observed in both subgroups of continuous infusion (*N*_CI_ = 65, *N*_II_ = 66, Fig. 3, RR 1.22, 95% CI 0.91–1.65) and extended infusion (*N*_EI_ = 357, *N*_II_ = 362, Fig. 3, RR 1.07, 95% CI 0.97–1.19).

### Microbiological cure rate

**Fig 4:**
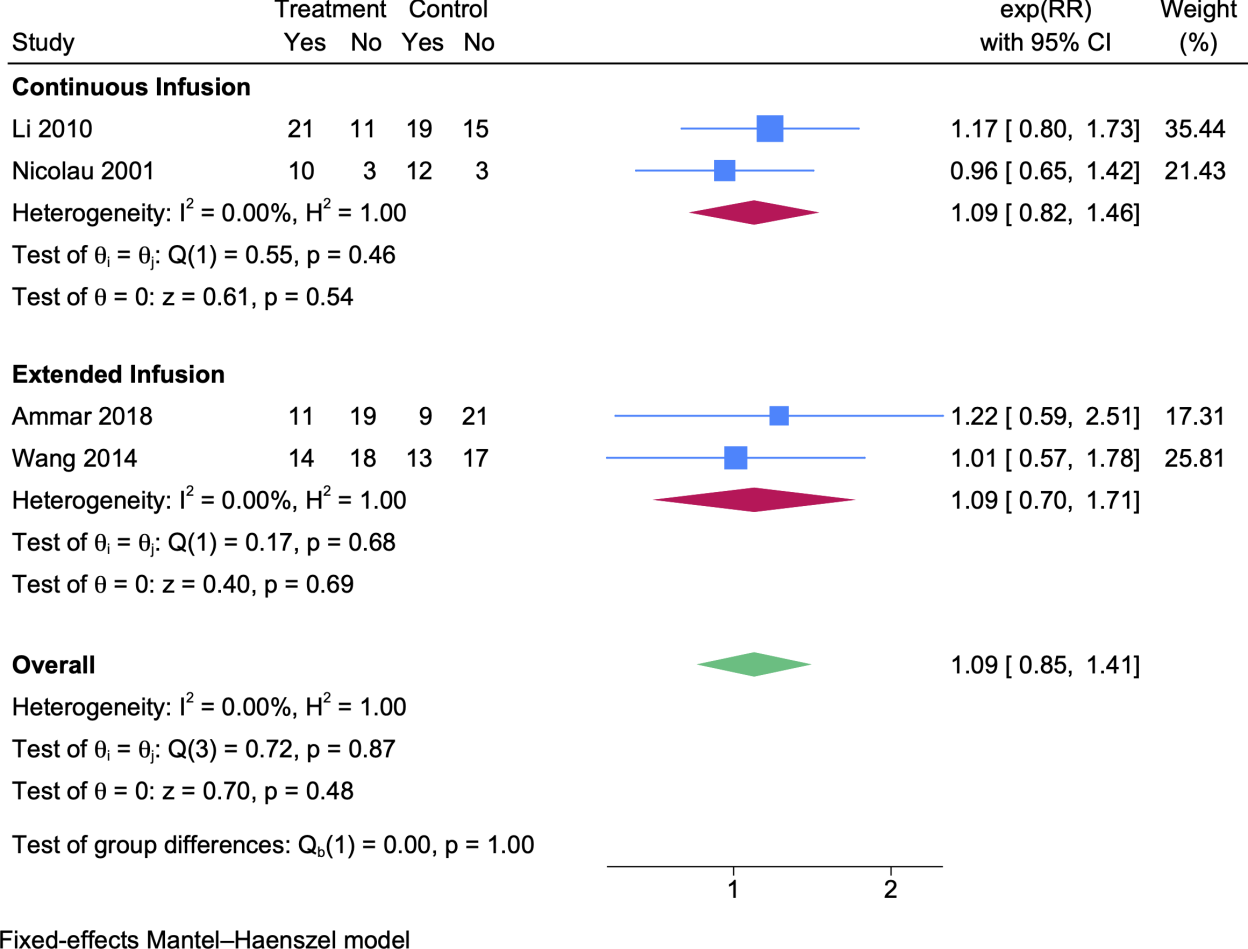
A forest plot representation of the comparison of the microbiological cure rates between prolonged infusion vs intermittent infusion of beta-lactam antibiotics.

For microbiological cure, four prolonged vs intermittent infusion RCTs (27, 28, 30, 36) were included. Compared to the intermittent group, the microbiological cure rate of the prolonged infusion group was higher but not significantly (Fig. 4, RR 1.09, 95% CI 0.85–1.41). The same trends were observed in both subgroups of continuous infusion (*N*_CI_ = 45, *N*_II_ = 49, Fig. 4, RR 1.09, 95% CI 0.82–1.46) and extended infusion (*N*_EI_ = 62, *N*_II_ = 60, Fig. 4, RR 1.09, 95% CI 0.7–1.71).

### Adverse events

**Fig 5:**
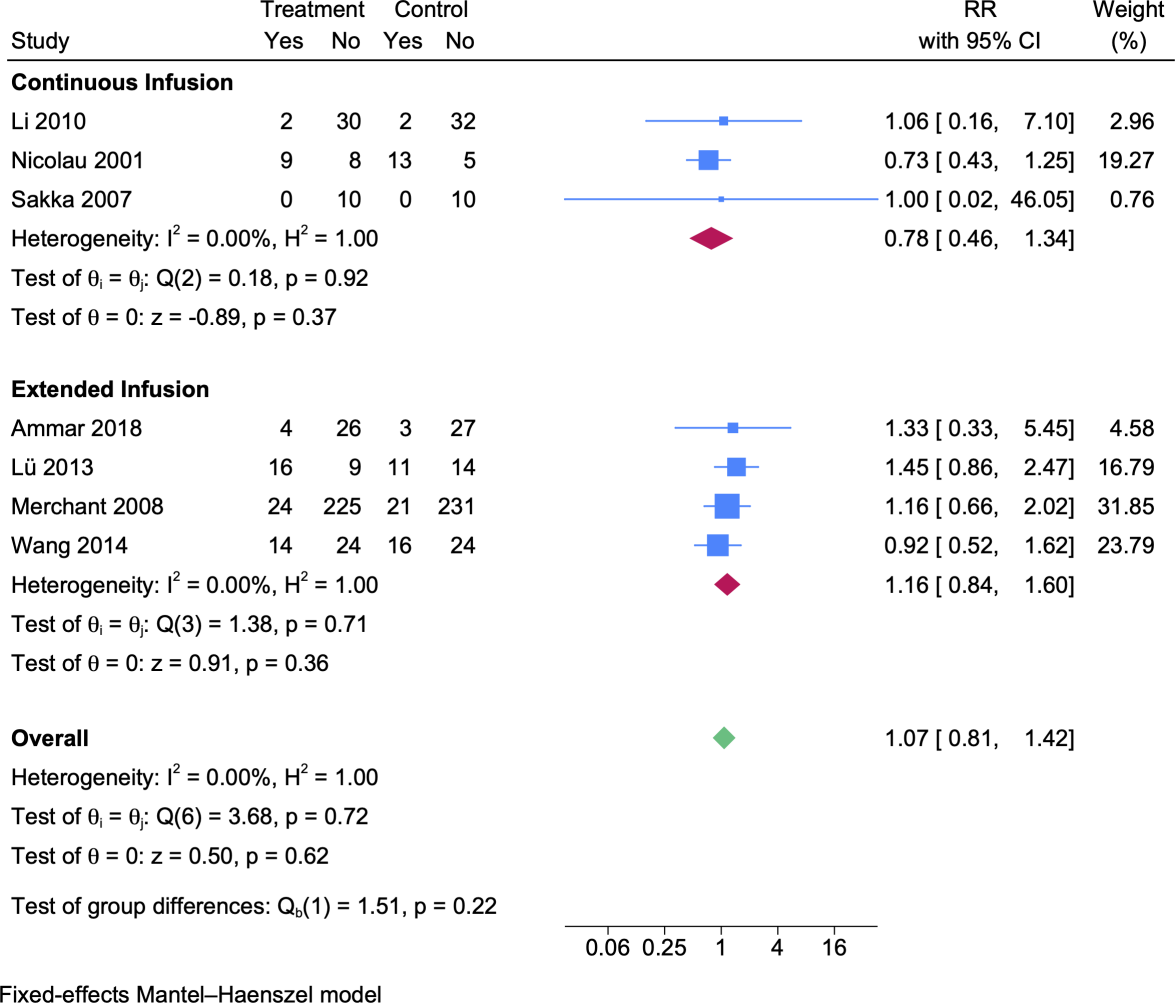
A forest plot representation of the comparison of adverse event rates between prolonged infusion vs intermittent infusion of beta-lactam antibiotics.

For adverse events, seven prolonged vs intermittent infusion RCTs (27–30, 33, 34, 36) were included. Compared to the intermittent infusion, the adverse event rate of the prolonged infusion group was higher but not significantly (Fig. 5, RR 1.07, 95% CI 0.81–1.42). In the continuous infusion subgroup, the adverse event rate was lower but not significantly (*N*_CI_ = 59, *N*_II_ = 62, Fig. 5, RR 0.78, 95% CI 0.46–1.34). In the extended infusion subgroup, the adverse event rate was higher but not significantly (*N*_EI_ = 342, *N*_II_ = 347, Fig. 5, RR 1.16, 95% CI 0.84–1.6).

### ICU length of stay

**Fig 6:**
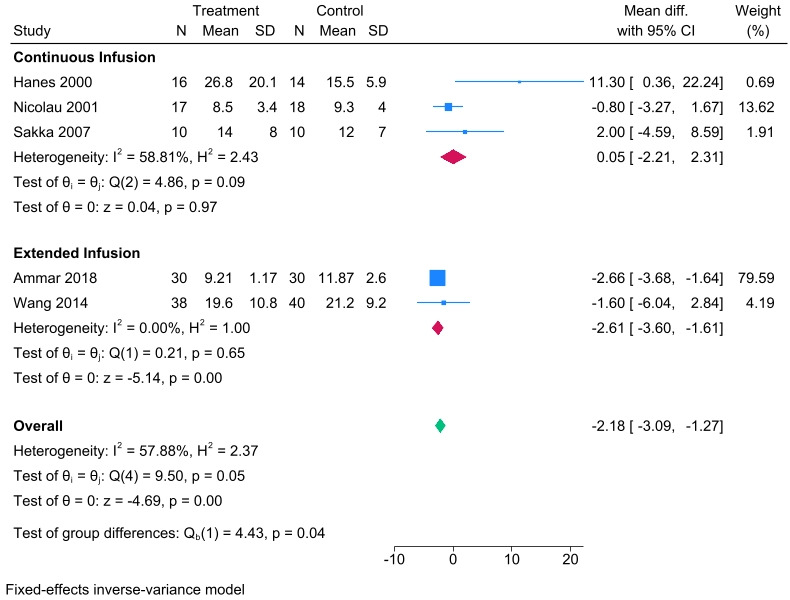
A forest plot representation of the comparison of the ICU length of stay between prolonged infusion vs intermittent infusion of beta-lactam antibiotics.

For ICU length of stay, five prolonged vs intermittent infusion RCTs (26, 28–30, 36) were included. Compared to the intermittent infusion group, the ICU length of stay was significantly shortened in the prolonged infusion group (Fig. 6, mean difference [MD] −2.18, 95% CI −3.09 to −1.27) and extended infusion subgroup (*N*_EI_ = 68, *N*_II_ = 70, Fig. 6, MD −2.61, 95% CI −3.6 to –1.61). However, no statistical difference in ICU length of stay was observed for subgroup continuous infusion (*N*_CI_ = 43, *N*_II_ = 42, Fig. 6, MD 0.05, 95% CI −2.21 to 2.31).

### Heterogeneity statistics

We analyzed the heterogeneity for each outcome according to the *I*^2^ statistic and *H*^2^ statistic; no significant heterogeneity was found in mortality (*I*^2^ = 0%, *H*^2^ = 1), microbiological cure rate (*I*^2^ = 0%, *H*^2^ = 1), and adverse events (*I*^2^ = 0%, *H*^2^ = 1). Moderate heterogeneities were observed among the included studies for clinical cure rate (*I*^2^ = 42.56%, *H*^2^ = 1.74) and ICU length of stay (*I*^2^ = 57.88%, *H*^2^ = 2.73).

## DISCUSSION

This meta-analysis compared the impact of prolonged infusion vs intermittent infusion administration on the clinical outcome of beta-lactam antibiotics in the treatment of adult ICU patients with pneumonia. The results suggest that compared to intermittent infusion, prolonged infusion, both continuous and extended infusion, of beta-lactam antibiotics does not show statistically significant benefits in terms of mortality, clinical cure rate, microbiological cure rate, and rate of adverse events. Only the prolonged infusion showed the advantage of shorter ICU length of stay compared with intermittent infusion. However, although statistical significance was not observed, clinical cure rate and microbiological cure rate were numerically higher for the prolonged infusion regimens, while mortality and rate of adverse events were lower. Perhaps these observations are not surprising, given the relatively lower number of eligible studies, most of which also have a relatively lower number of subjects enrolled.

Lal et al. (25) published a similar meta-analysis in 2016, which is the only previous meta-analysis that compared the prolonged and intermittent beta-lactam infusion for the treatment of pneumonia. They reported that there was no statistically significant difference between groups of prolonged and intermittent infusion in mortality, microbiological cure rate, and adverse events, which is consistent with our analysis results. However, there are some differences worth highlighting. Their analysis reported that the clinical cure rate is obviously higher among patients who received extended or continuous infusion than those who received intermittent infusion (OR 2.45, 95% CI, 1.12–5.37). However, our study reported that the difference was not statistically significant in clinical cure rate, only a slight advantage of prolonged infusion (RR 1.09, 95% CI, 0.99–1.21). In addition, we supplemented the analysis with ICU length of stay, which showed an advantage of prolonged infusion compared to intermittent infusion groups. We also analyzed the subgroups continuous infusion vs intermittent infusion and extended infusion vs intermittent infusion separately. Lal et al.’s study included a variety of reports of experimental designs, including retrospective and prospective studies, rather than just RCTs. The most recent experimental data they included was published in 2012, and some RCTs have been conducted since then. Their study did not consider the publications in non-English language. In addition, they only included nosocomial pneumonia patients, and there was no mention of patients with severe community-acquired pneumonia admitted to ICUs.

A meta-analysis by Lee et al. (37) in 2018 reported that, when comparing continuous infusion vs intermittent bolus of beta-lactam antibiotics in critically ill patients with respiratory infections, continuous infusion was associated with a greater clinical cure (RR 1.177, 95% CI 1.065–1.3), with no difference in mortality between continuous infusion and intermittent bolus (RR 0.845, 95% CI 0.644–1.108). Our meta-analysis observed a similar point estimate for lower mortality with prolonged infusion, but no difference in clinical cure between continuous and intermittent infusion groups. Aboulatta et al. (38) compared the extended vs intermittent infusion of beta-lactams in critically ill patients with respiratory infections. The meta-analysis showed lower mortality of extended infusion (risk differences [RD] 0.1, 95% CI 0.15–0.04), and no significant difference between extended and intermittent infusion of clinical cure (RD 0.11, 95% CI −0.09 to 0.3) and ICU length of stay (RD −2.37, 95% CI −5.17 to 0.42) was observed. Our meta-analysis also showed no difference between extended and intermittent infusion of the clinical cure rate in ICU patients with pneumonia, which is consistent with Aboulatta et al.’s report. However, while no significant mortality benefit was observed, the extended infusion group demonstrated a shorter ICU length of stay. Extended infusion of beta-lactam antibiotics provided a greater improvement in primary clinical outcomes in ICU patients with respiratory infections compared to those with pneumonia.

Some RCTs on sepsis included patients with pneumonia; however, their data did not distinguish pneumonia patients separately (39–43). BLING III, a recently published international, large-scale RCT, compared beta-lactam antibiotics administered via continuous and intermittent infusion in patients with sepsis (44). This study found no advantage of continuous infusion over intermittent infusion in terms of 90-day mortality or ICU length of stay. However, continuous infusion was related to a higher clinical cure rate. Adverse events occurred in both groups (0.3% in CI vs 0.2% in II), with one serious case in the continuous infusion group, which was potentially linked to higher meropenem concentrations. Due to the lack of data on pneumonia patients, we excluded the BLING III study. Abdul-Aziz et al. recently compared the prolonged infusion and intermittent infusion of beta-lactam antibiotics in adults with sepsis or septic shock in a systematic review and meta-analysis, including patients with lung infections. Prolonged infusion was associated with lower 90-day mortality compared to intermittent infusion. However, no significant difference was observed between prolonged infusion and intermittent infusion groups among patients with lung infections, which aligns with our findings in pneumonia patients. Prolonged infusion also showed an advantage in ICU mortality and clinical cure rate. No differences were observed between prolonged infusion and intermittent infusion regarding microbiological cure rate, occurrence of adverse events, or ICU length of stay (45). Our analysis found no statistically significant difference between prolonged infusion and intermittent infusion groups in clinical cure rate, microbiological cure rate, or occurrence of adverse events. However, prolonged infusion was associated with a shorter ICU length of stay than intermittent infusion. In addition, an RCT systematic review and meta-analysis reported optimized mortality (RR 0.79, 95% CI 0.64–0.98) and microbiological cure rate (RR 1.16, 95% CI 1.03–1.29) for critically ill patients on continuous infusion compared with those on intermittent infusion of beta-lactam antibiotics. There was no difference in ICU length of stay between the two infusion groups (MD 0.03, 95% CI −1.44 to 1.5 days) (46). Reviews of critically ill sepsis patients by Chen et al. (47) and Roberts et al. (48) suggested significant advantages of mortality and clinical cure rates. However, our meta-analysis showed no significant advantages in mortality, clinical cure rates, and microbiological cure rates of continuous infusion in ICU pneumonia patients. Consistent with our result, no significant difference in mortality of prolonged infusion was observed in the meta-analysis of Chant et al. (49).

Findings from our analyses showed that there was only a slight mortality benefit for prolonged infusion, including both continuous infusion and extended infusion, compared with intermittent infusion for ICU patients with pneumonia. However, clinically, there is a better outcome in continuous infusion with no significant difference in survival compared to intermittent infusion. There are also cases of treatment failure and death in patients who have reached their treatment goal. Pneumonia in ICU patients is very difficult to treat and may be highly correlated with Sequential Organ Failure Assessment (SOFA) score rather than antibiotic regimens (29). Felton et al. mimicked PK profiles of piperacillin-tazobactam by a hollow-fiber infection model with *Pseudomonas aeruginosa* and compared the impact of bolus dosing vs continuous infusion. The results showed that the bacterial killing effects were similar in two infusion methods. In addition, high initial bacterial densities were associated with progressive growth of drug-resistant subpopulations, which was not found to be the case with low initial bacterial densities (50).

This study is the updated systematic review and meta-analysis of RCTs comparing the beta-lactam antibiotics treatment clinical outcomes following prolonged infusions vs intermittent infusions in ICU pneumonia patients from 2016 and has key strengths with additional new and updated analyses. We conducted subgroup meta-analyses of continuous vs intermittent infusion and extended vs intermittent infusion, which had not been reported previously. In addition, previously published reviews usually limited the language of publication to English, unlike this current study, where we considered all studies and included several new studies published in the Chinese language that had not been included before in previous reviews. However, this meta-analysis has some limitations. First, for the overall assessment of the five domains considered, all included studies were considered to have some concerns. Second, the most recent study included was published in 2018. Given the significant changes in antibiotics use patterns, PK/PD target recommendations, and the prevalence of antimicrobial resistance over the past several years, it is possible that the inclusion of more recent data could have influenced the findings. Moreover, there are several ongoing large sample size research that could not be included at this stage, which could have improved the study’s findings (supplementary information S4 at GitHub). Third, prolonged infusion administration strategies and the type of beta-lactam antibiotics used in the trials differed in each study. The definitions of mortality, clinical cure, microbiological cure, or adverse events were also not entirely consistent from study to study. Finally, for some endpoints, missing data may lead to inaccuracies in meta-analysis results. Despite the above limitations, this study gives a comprehensive assessment of the comparison of continuous or extended vs intermittent infusion dosing of beta-lactam antibiotics in ICU patients with pneumonia.

In conclusion, in the treatment of ICU patients with pneumonia, prolonged infusion of beta-lactam antibiotics was associated with a significantly shorter ICU length of stay. No significant advantage was observed for prolonged infusion, both extended infusion and continuous infusion, in terms of mortality, clinical cure, or microbiological cure in ICU patients with pneumonia. However, adequately powered RCTs of ICU patients with pneumonia are required for a definitive conclusion of the role of prolonged infusion of beta-lactam antibiotics in this special patient population.

## MATERIALS AND METHODS

This systematic review was conducted according to the PRISMA 2020 statement (51).

### Eligibility criteria

Inclusion criteria were as follows: (i) RCTs comparing continuous or extended vs intermittent infusions of one or more beta-lactam antibiotics; (ii) population is adult (≥18 years old) patients with pneumonia receiving care in the ICU; (iii) intervention is prolonged infusion; (iv) comparator is intermittent infusion.

#### Definition

1. Pneumonia: accepted all definitions at the time of patient recruitment (supplementary information S1 at GitHub).
2. Outcomes: accepted all definitions of mortality, clinical cure, microbiological cure, and adverse events (supplementary information S2 at GitHub).
3. ICU patients were defined as the patients recruited in an ICU, or the inclusion criteria described such that the patients would normally be managed in an ICU (e.g., patients receiving invasive mechanical ventilation), or the patients had an average ICU length of stay of ≥2 days, or a majority of the patients received a therapy that is delivered in the ICU (e.g., invasive mechanical ventilation), or a severity of illness score that reflected a critically ill population.
4. Prolonged infusion includes continuous infusion and extended infusion. Continuous infusion was defined as a constant rate of intravenous antibiotic administration either as a sequential 6 hours, 8 hours, 12 hours, or 24 hours infusion. Extended infusion was defined as intravenous antibiotic administration over a duration of ≥2 hours during a dosing interval but not over the entire dosing interval.
5. Intermittent infusion was defined as administration of an intravenous antibiotic infusion for <2 hours.

### Search strategy

Medline (via PubMed), CINAHL, EMBASE, Cochrane Central Register of Controlled Trials (CENTRAL), and ClinicalTrials.gov were searched to identify eligible trials to be included for review (22 November 2023). The search was performed with no restrictions on language, publication date, or publication status. This search was supplemented by a manual review of the reference citation of published systematic reviews comparing prolonged (including continuous and extended) vs intermittent infusion of beta-lactam antibiotics (keywords are described in supplementary information S3 at GitHub).

### Study selection

Two reviewers (YL and FBS) independently selected the study according to the inclusion and exclusion criteria. All references were managed by Covidence systematic review software (Veritas Health Innovation, Melbourne, Australia) and EndNote 20 (Clarivate Analytics, Philadelphia, PA, USA). All excluded studies and the reason for exclusion were attached in supplementary information S4 at GitHub.

### Data extraction

Two reviewers (YL and FBS) independently screened all studies by title and abstract. Two reviewers (YL and FBS) reviewed all full-text studies according to selection criteria, set up a sample form of data extraction, and extracted data. The third reviewer (MHAA) checked these independently. The following data were extracted:

- Study characteristics: year of publication, study design, study period, recruiting countries, and number of patients enrolled.
- Participant characteristics: age, sex, severity of illness scores at baseline (APACHE II), and definition of pneumonia.
- Study intervention and comparator details: antibiotics and dosing regimen.
- Primary outcome: mortality (definitions in supplementary information S2 at GitHub).
- Secondary outcomes: clinical cure rate, ICU length of stay, microbiological cure rate, adverse events, and PK/PD target attainment.

### Quality assessment

Two reviewers (YL and FBS) independently assessed the risk of bias of all outcomes. Disagreements were resolved by discussion between the two reviewers. The quality of each included study was assessed using the Cochrane risk-of-bias tool for randomized trials (RoB 2; outcomes were attached in supplementary information S5 at GitHub) (52).

### Statistical analysis

For the primary outcome and the secondary outcomes of clinical cure rate, microbiological cure rate, and adverse events, the RR was estimated with a 95% CI. For ICU length of stay, the MD was estimated with a 95% CI. *I*^2^ statistic and *H*^2^ statistic were used to quantify the heterogeneity of the RR and MD among studies. The heterogeneity was described as low, moderate, and high relative to *I*^2^ values of 25%, 50%, and 75% respectively. A value of *H*^2^ = 1 indicates perfect homogeneity among the studies. The fixed effect model was used to perform meta-analysis on mortality, clinical cure rate, microbiological cure rate, adverse events, and ICU length of stay. Forest plots were used in the meta-analysis to illustrate the estimates, 95% CI, and the weight of each estimate. Funnel plots and Egger’s test were used to illustrate the publication bias; for all analyses, *P* < 0.05 was considered significant (53). All meta-analyses were conducted in STATA (StataCorp. 2023. Stata Statistical Software: Release 18. College Station, TX: StataCorp LLC.).
